# A cross-sectional analysis of the association between physical activity, depression, and all-cause mortality in Americans over 50 years old

**DOI:** 10.1038/s41598-022-05563-7

**Published:** 2022-02-10

**Authors:** Jose Luis Perez-Lasierra, Belén Moreno-Franco, Alejandro González-Agüero, Elena Lobo, Jose A. Casajus

**Affiliations:** 1 Department of Physiatry and Nursing, Universidad de Zaragoza, 50009 Zaragoza, Spain; 2 GENUD (Growth, Exercise, Nutrition and Development) Research Group, 50009 Zaragoza, Spain; 3 EXERNET Red de Investigación en Ejercicio Físico y Salud, Zaragoza, Spain; 4 Department of Microbiology, Pediatrics, Radiology and Public Health, Universidad de Zaragoza, 50009 Zaragoza, Spain; 5 Instituto de Investigación Sanitaria Aragón, Hospital Universitario Miguel Servet, 50009 Zaragoza, Spain; 6 CIBERCV Instituto de Salud Carlos III, 28029 Madrid, Spain; 7 CIBERSAM Instituto de Salud Carlos III, 28029 Madrid, Spain; 8 Preventive Medicine and Public Health, Universidad de Zaragoza, C/Domingo Miral s/n, 50009 Zaragoza, Spain

**Keywords:** Depression, Lifestyle modification, Epidemiology

## Abstract

Depression is estimated to be the second leading cause of disability in the United States and is associated with a 52% increased risk of death. Lifestyle components may have an important role in depression pathogenesis. The aims of this study were to analyze the association of meeting the physical activity (PA) recommendation guidelines and depression, and to analyze the all-cause mortality risk of the joint association of PA and depression. This cross-sectional study included 7201 participants from the 2007–2014 National Health and Nutrition Examination Survey aged ≥ 50 years and linked to National Death Index records through December 31, 2015. Depression was defined as a score ≥ 10 using the Patient Health Questionnaire (PHQ-9). PA was self-reported, and total PA was used to classify participants as more active (≥ 600 MET-min/week) or less active (< 600 MET-min/week). The odds ratios for depression were examined according to be more active or less active. The hazard ratios (HR) for the association of PA level and depression status with all-cause mortality were examined. Being more active was associated with reduced odds for depression. Compared with less active participants with depression, those who were more active and having depression had HR 0.45 (95% CI 0.22, 0.91, p = 0.026) for all-cause mortality. Being more active is associated with lower odds for depression and seems to be a protective factor against the increased all-cause mortality risk due to depression.

## Introduction

Depression is a common mental disorder affecting more than 264 million people worldwide^1^. In the United States, it is estimated to be the second cause of disability^2^, with an increasing trend in non-institutionalized population, mainly in older people^3^.

People suffering from depression usually show different neurovegetative, neurocognitive, and emotional symptoms^4^. Among them are low levels of mood, anhedonia, feelings of worthlessness or guilt, fatigue or loss of energy, disruptive appetite, sleep disturbance, and difficulty to think or concentrate^4^, which frequently lead to decreased quality of life, disability, suicide ideation, or even suicide attempts^5,6^.

Lifestyle components may have an important role in depression pathogenesis. Unhealthy behaviors such as sedentarism, physical inactivity, poor diet, or substance abuse have been associated with a significantly higher risk of depression^7,8^, contrary to the protective effect of a healthy lifestyle^8^. Physical activity (PA), defined as any bodily movement produced by skeletal muscles that requires energy expenditure^9^, is a cornerstone in the primary prevention of chronic diseases, including depression^8,10,11^. Moreover, evidence also suggests that PA is a recognized strategy in secondary prevention^12^ and that PA plays a significant role concerning late-life depression consequences, such as suicide^13^. Biological theories about the antidepressant mechanisms of PA are mainly based on the improvement of neuroplasticity, and in the reduction of inflammation and oxidative stress, while psychosocial theories are based on the improvement of self-esteem, social support, and self-efficacy^14^.

Although the benefits of PA against depression are well documented^8,10,11^, few studies have analyzed whether compliance with PA recommendations^9^ is enough to obtain a preventive effect against depression^10^. Furthermore, those studies are mainly focused on young or middle-aged women, health care workers, or college students^10^, and only two studies are focused on older people^15,16^. On the contrary, the protective effect of complying with PA recommendations against all-cause mortality in the general population is widely known^9,17^. Evidence also suggests that depression is associated, by itself, with a higher mortality risk, reaching a 52% increased risk of death^18^. Despite the evidence of these associations with mortality, to our knowledge, no study has analyzed the all-cause mortality risk of the joint association of PA and depression in older adults. Therefore, the purpose of this study was to analyze the association of meeting the PA recommendations and depression and to analyze the all-cause mortality risk of the joint association of PA and depression in non-institutionalized, older American adults.

## Methods

### Study design and population

The National Health and Nutrition Examination Survey (NHANES), conducted by the National Center for Health Statistics (NCHS), is an annual national cross-sectional survey of a representative sample of non-institutionalized United States population. The survey uses a stratified, multistage sample design to randomly select approximately 7000 residents across the country each year. Participation in the survey is confidential and voluntary. Public-use linked mortality files from the National Death Index (NDI) are available for continuous NHANES 1999–2014, providing mortality data from the date of survey participation through December 31, 2015.

The present study used data from 4 cross-sectional NHANES waves conducted from 2007 to 2014 and their linked mortality files. Details about linkage of NHANES data with NDI records have been published elsewhere^19^. For this analysis, sample was reduced to participants ≥ 50 years old who were followed up for mortality outcomes ≥ 12 months after the enrollment in the study to minimize bias from reverse causation (n = 10,908). Participants with missing data on PA (n = 2523), depression (n = 811), and other covariables (n = 373) were excluded, so the final sample included 7201 participants.

All participants provided written informed consent, and all methods were carried out in accordance with relevant guidelines and regulations. The Ethics Review Board of the NCHS approved measurement procedures, data collection, and posting of the data online for public use.

### Definition and assessment of depression

Depression was assessed by means of the Patient Health Questionnaire-9 (PHQ-9), a widely-used self-report depression screener that consists of 9 items to assess depressive symptoms over the last 2 weeks^20^. The PHQ-9 score can range from 0 to 27, since each of the 9 items can be scored from 0 (not at all) to 3 (nearly every day)^20^. Scores ≥ 10 represent clinically significant depressive symptoms^21^, so for this study, depression has been defined as score ≥ 10 in the PHQ-9. This is a common cut-point that has been used in previous studies^22^ and maximized combined sensitivity and specificity^23^.

### Assessment of physical activity

PA was assessed by interview using the Global Physical Activity Questionnaire (GPAQ) created by the World Health Organization (WHO)^24^. This questionnaire analyzes the usual PA performed in a typical week in 3 different domains (PA at work/domestic, PA in transport/travel, and PA in leisure time), as long as it has been carried out in continuous periods of 10 min. The questionnaire also considers the intensity at which it has been performed (moderate or vigorous). The total metabolic equivalent per minute per week (MET-min/week) was calculated following the GPAQ protocol^25^.

Based on PA recommendation guidelines by the WHO^9^, the subjects were classified into two different groups. Those who performed at least 150 min of moderate to vigorous PA (≥ 600 MET-min/week) and met the PA recommendations for adults compose the more-active group, and those who performed less than 150 min of moderate to vigorous PA (< 600 MET-min/week) and thus did not meet the recommendations, composed the less-active group.

### Mortality

Survival time was counted from the date of survey participation to the date of death or the end of the study follow-up period (December, 31, 2015), whichever came first. In this study all-cause mortality was used as the main outcome for mortality, classifying participants as alive or deceased.

### Assessment of additional covariates

Demographic, lifestyle, anthropometric, and health data were obtained and used to adjust the results of regression models. The selection of these specific variables was based on their possible confounding role in the associations analyzed^6,26^.

Demographics included age (50–59; 60–69; 70–79; ≥ 80), sex, race/ethnicity (Mexican American, Other Hispanic, Non-Hispanic White, Non-Hispanic Black, and Other (including Multi-Racial)), annual household income (0–19,999 USD; 20,000–44,999 USD; 45,000–74,999 USD, and ≥ 75,000 USD), and educational level (less than 9th grade; 9th–12th grade, no diploma; high school graduate; college or Associate´s degree; and college graduate or above).

Lifestyle risk factors included alcohol consumption in the last 12 months classified as 0 drinks/day, < 2 drinks/day, and ≥ 2 drinks/day, and smoking status was defined as never smoked, former smoker, and smoker.

Anthropometric included body mass index (BMI), calculated as weight in kilograms divided by height in meters squared and classified in < 25.0 kg/m^2^, 25.0–29.9 kg/m^2^, and ≥ 30.0 kg/m^2^. Self-reported medical diagnosis of hypertension, dyslipidemia, or type 1 and 2 diabetes, or self-reported use of antihypertensive medication, lipid-lowering drugs, or hypoglycemic medication, were used to classify participants as having arterial hypertension, dyslipidemia, and diabetes, respectively.

### Statistical analysis

According to the NHANES analytical guidelines, all data were downloaded, merged, and analyzed, incorporating appropriated combined weights, primary sampling unit, and strata provided by NHANES^27^. Moreover, public-use linked mortality files from NDI were merged with NHANES data following the appropriate guidelines^19^.

Categorical variables were expressed as frequency (%), and continuous variables were presented as mean and standard error (SE). Descriptive analyses were carried out for the overall samples and divided by PA groups. Logistic regressions models according to the PA group of the participants were conducted to examine the adjusted odds ratios (OR) for depression. The first model was unadjusted, and the second only age-adjusted. Model A was adjusted by age, sex, race/ethnicity, annual household income, and educational level. Model B was additionally adjusted by smoking status, alcohol consumption, BMI, arterial hypertension, dyslipidemia, and diabetes.

Cox proportional hazards regression models were performed to examine hazard ratios (HRs) and 95% CIs for the association between PA level and depression status with all-cause mortality. Furthermore, adjusted survival curves were plotted. When the PA level and depression status joint association with all-cause mortality was analyzed, the less-active (< 600MET-min/week) and with-depression (PHQ-9 ≥ 10) subgroup was considered the reference group when hazard ratios for the three other subgroups were calculated. In this case, the model was adjusted for potential confounders, including age at baseline, sex, race/ethnicity, annual household income, educational level, smoking status, alcohol consumption, BMI, arterial hypertension, dyslipidemia, and diabetes. The proportional hazards assumption was not violated as examined by log–log survival plots and correlations of follow-up time and Schoenfeld residuals from the adjusted Cox models^28^.

A two-sided p-value of 0.05 was considered statistically significant. Statistical analysis was performed using SPSS statistical software (ver. 24.0 IBM Corp., Armonk, NY, USA) and R statistical software (ver. 4.0.4).

## Results

The overall prevalence of depression in ≥ 50-years-old non-institutionalized Americans was 7.8%. The prevalence among those who met and did not meet the PA recommendations for adults was 5.3% and 11.0%, respectively (Table 1). The more-active group had a lower prevalence of obesity, hypertension, dyslipidemia, and diabetes, as well as higher educational level and annual household income than the less-active group (Table 1).

**Table 1:** Baseline characteristics of study participants according to level of physical activity. Data are expressed as weighted percentages and unweighted number of participants for categorical variables, and as weighted mean (standard error) for continuous variables. Less active: performed < 600 MET-min/week; More active: performed ≥ 600 MET-min/week; Other: other race, including Multi-Racial; AA degree: Associate’s degree.

	Overall	Less active	More active	p value for interaction
N, %	100 (7201)	43.1 (3439)	56.9 (3762)	**< 0.001**
Depression, %	7.8 (676)	11.0 (437)	5.3 (239)	**< 0.001**
PHQ-9 Score, points	2.92 (0.07)	3.62 (0.11)	2.38 (0.08)	**< 0.001**
Deaths, %	6.4 (655)	9.4 (428)	4.1 (227)	**< 0.001**
**Age, %**				
50–59	43.5 (2403)	37.4 (972)	48.1 (1431)	**< 0.001**
60–69	32.2 (2542)	32.0 (1201)	32.4 (1341)	
70–79	16.5 (1501)	19.5 (809)	14.2 (692)	
> 80	7.8 (755)	11.1 (457)	5.4 (298)	
Male, %	49.7 (3829)	41.7 (1601)	55.8 (2228)	**< 0.001**
**Race/ethnicity, %**				
Mexican American	3.8 (765)	4.3 (386)	3.4 (379)	**< 0.001**
Other Hispanic	3.2 (660)	3.6 (323)	2.8 (337)	
Non-Hispanic White	80.1 (3769)	77.6 (1727)	82.0 (2042)	
Non-Hispanic Black	9.0 (1581)	10.7 (823)	7.7 (758)	
Other	3.9 (426)	3.7 (180)	4.0 (246)	
**Annual household income, %**				
0–19,999 USD	13.6 (1592)	17.7 (887)	10.5 (705)	**< 0.001**
20,000–44,999 USD	28.2 (2405)	31.4 (1225)	25.8 (1180)	
45,000–74,999 USD	21.4 (1376)	19.9 (607)	22.6 (769)	
≥ 75,000 USD	36.7 (1828)	31.0 (720)	41.1 (1108)	
**Educational level, %**				
Less than 9th Grade	4.9 (755)	7.0 (442)	3.4 (313)	**< 0.001**
9th–12th Grade, No diploma	10.3 (1038)	12.9 (596)	8.4 (442)	
High School Graduate	22.4 (1656)	24.0 (820)	21.2 (836)	
College or AA Degree	30.0 (2009)	29.7 (903)	30.2 (1106)	
College Graduate or Above	32.3 (1743)	26.4 (678)	36.7 (1065)	
**Alcohol consumers, %**				
0 drinks/day	24.1 (2213)	28.9 (1232)	20.5 (981)	**< 0.001**
< 2 drinks/day	67.8 (4490)	64.8 (2022)	70.1 (2468)	
≥ 2 drinks/ day	8.1 (498)	6.3 (185)	9.4 (313)	
**Smoking status, %**				
Never	45.1 (3102)	43.6 (1431)	46.3 (1671)	**0.046**
Former	38.6 (2845)	38.5 (1367)	38.7 (1478)	
Smoker	16.2 (1254)	17.9 (641)	15.0 (613)	
**BMI, %**				
< 25	25.3 (1750)	21.5 (745)	28.1 (1005)	**< 0.001**
25–29.9	35.6 (2533)	31.3 (1110)	38.9 (1423)	
≥ 30	39.1 (2918)	47.1 (1584)	33.0 (1334)	
Arterial hypertension, %	51.1 (4056)	57.5 (2136)	46.2 (1920)	**< 0.001**
Dyslipidemia, %	56.1 (4067)	58.7 (2015)	54.1 (2052)	**0.003**
Diabetes, %	16.8 (1573)	21.9 (912)	12.8 (661)	**< 0.001**

Significant values are in bold.

The likelihood of having depression was lower for those participants in the more-active group compared to those in the less-active group. The weighted odds for having depression after adjusting the results by age, sex, race/ethnicity, annual household income, educational level, alcohol consumption, smoking status, BMI, arterial hypertension, dyslipidemia, and diabetes were 0.57 (95% CI 0.44, 0.72, p < 0.001) for the more-active group compared to the less-active group (Table 2). Additionally, Supplementary Table 1 includes tests of the weighted odds for having depression among three PA levels subgroups: < 600 MET-min/week, 600–1200 MET-min/week, and > 1200 MET-min/week.

**Table 2:** Odds ratio (95% CI) for depression according to physical activity levels. Data are representative of non-institutionalized American population. Model A is adjusted by age, sex, race/ethnicity, annual household income, and educational level. Model B is additionally adjusted by alcohol consumption, smoking status, BMI, arterial hypertension, dyslipidemia, and diabetes. ^a^Significant differences between Less-active and More-active groups.

	Physical activity level (MET-min/week)		*p-*value
	Less active (< 600)	More active (≥ 600)	
Unadjusted	1.00 (ref)^a^	0.46 (0.36, 0.57)	< 0.001
Age-adjusted	1.00 (ref)^a^	0.41 (0.33, 0.52)	< 0.001
Multivariable-adjusted Model A	1.00 (ref)^a^	0.52 (0.41, 0.65)	< 0.001
Multivariable-adjusted Model B	1.00 (ref)^a^	0.57 (0.44, 0.72)	< 0.001

In addition, if the total PA was divided according to the different domains analyzed (PA at work/domestic, PA in leisure time, and PA in transport/travel), only those who performed ≥ 600 MET-min/week of leisure-time PA had significantly lower odds for having depression compared to those who performed < 600 MET-min/week of leisure-time PA (OR 0.47, 95% CI 0.32, 0.67, p < 0.001) (Supplementary Table 2).

During a median 54.0 months (interquartile range 12–108 months) of follow-up, 655 deaths occurred among 7201 individuals in the study. The percentage of deaths among those who met and did not meet the PA recommendations for adults were 4.1% and 9.4%, respectively (Table 1). Moreover, the percentage of deaths in participants with and without depression were 9.4% and 6.1%, respectively.

**Figure 1:**
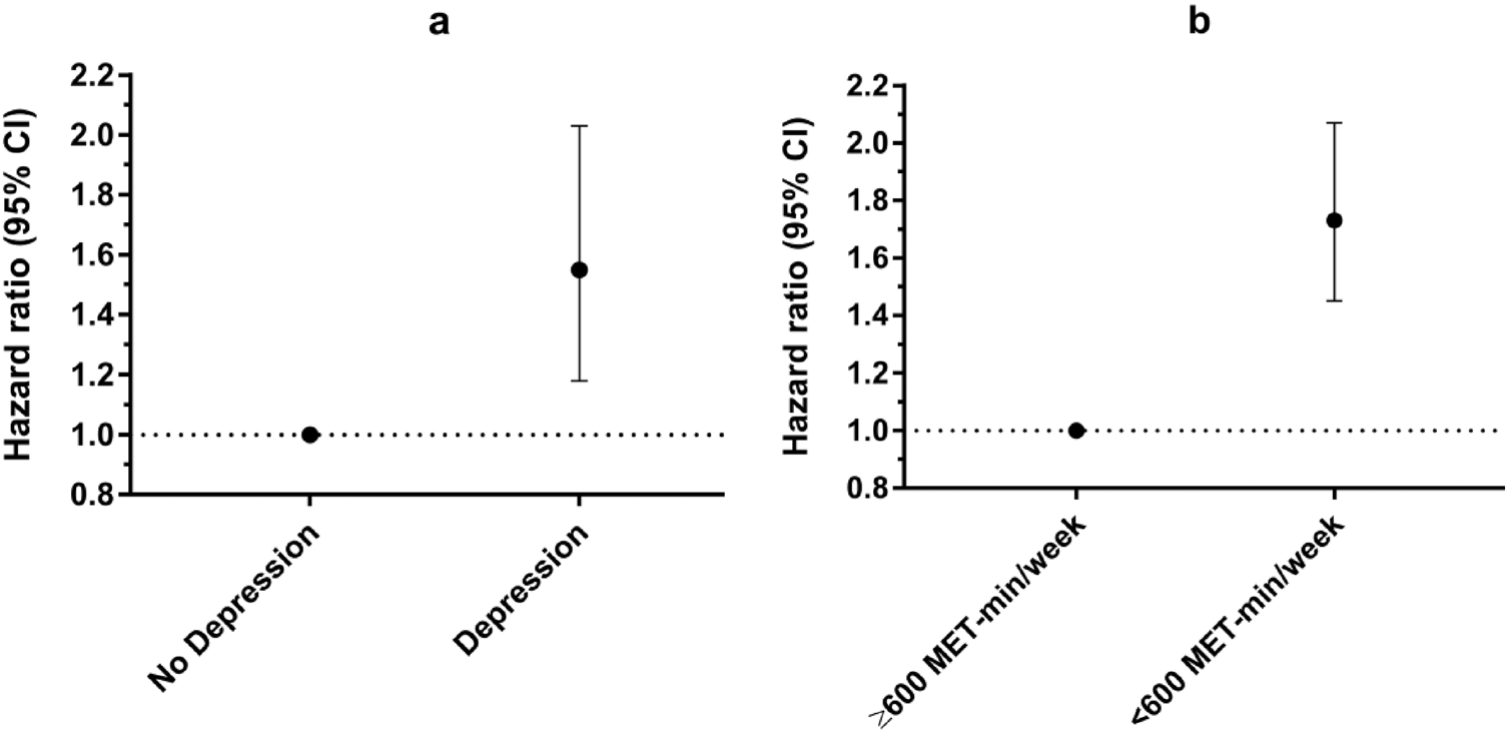
Hazard ratio (95% CI) for all-cause mortality according to (**a**) depression status, and (**b**) physical activity level. Data are representative of non-institutionalized American population. All hazard ratios were adjusted by age, sex, race/ethnicity, annual household income, educational level, alcohol consumption, smoking status, body mass index, arterial hypertension, dyslipidemia, and diabetes.

When studying HRs for all-cause mortality, those with depression had a 1.55-fold increased HR of death (95% CI 1.18, 2.03, p = 0.002) compared to those without depression (Table 3 and Fig. 1a). Moreover, those who performed < 600 MET-min/week had a 1.73-fold increased HR of death (95% CI 1.45, 2.07, p < 0.001) compared to those who performed ≥ 600 MET-min/week (Table 4 and Fig. 1b). These HRs were adjusted by age, sex, race/ethnicity, annual household income, educational level, alcohol consumption, smoking status, BMI, arterial hypertension, dyslipidemia, and diabetes.

**Table 3:** Hazard ratio (95% CI) for all-cause mortality according to depression status. Data are representative of non-institutionalized American population. Model A is adjusted by age, sex, race/ethnicity, annual household income, and educational level. Model B is additionally adjusted by alcohol consumption, smoking status, BMI, arterial hypertension, dyslipidemia, and diabetes. With depression: scored ≥ 10 in PHQ-9; Without depression: scored < 10 in PHQ-9. ^a^Significant differences between without-depression and with-depression groups.

	Depression status		*p-*value
	Without depression	With depression	
Unadjusted	1.00 (ref)^a^	1.72 (1.28, 2.30)	0.001
Age-adjusted	1.00 (ref)^a^	2.35 (1.75, 3.16)	< 0.001
Multivariable-adjusted Model A	1.00 (ref)^a^	1.88 (1.40, 2.51)	< 0.001
Multivariable-adjusted Model B	1.00 (ref)^a^	1.55 (1.18, 2.03)	0.002

**Table 4:** Hazard ratio (95% CI) for all-cause mortality according to physical activity level. Data are representative of non-institutionalized American population. Model A is adjusted by age, sex, race/ethnicity, annual household income, and educational level. Model B is additionally adjusted by alcohol consumption, smoking status, BMI, arterial hypertension, dyslipidemia, and diabetes. ^a^Significant differences between Less-active and More-active groups.

	Physical activity level (MET-min/week)		*p-*value
	More active (≥ 600)	Less active (< 600)	
Unadjusted	1.00 (ref)^a^	2.52 (2.07, 3.06)	< 0.001
Age-adjusted	1.00 (ref)^a^	1.91 (1.58, 2.30)	< 0.001
Multivariable-adjusted Model A	1.00 (ref)^a^	1.85 (1.53, 2.24)	< 0.001
Multivariable-adjusted Model B	1.00 (ref)^a^	1.73 (1.45, 2.07)	< 0.001

**Figure 2:**
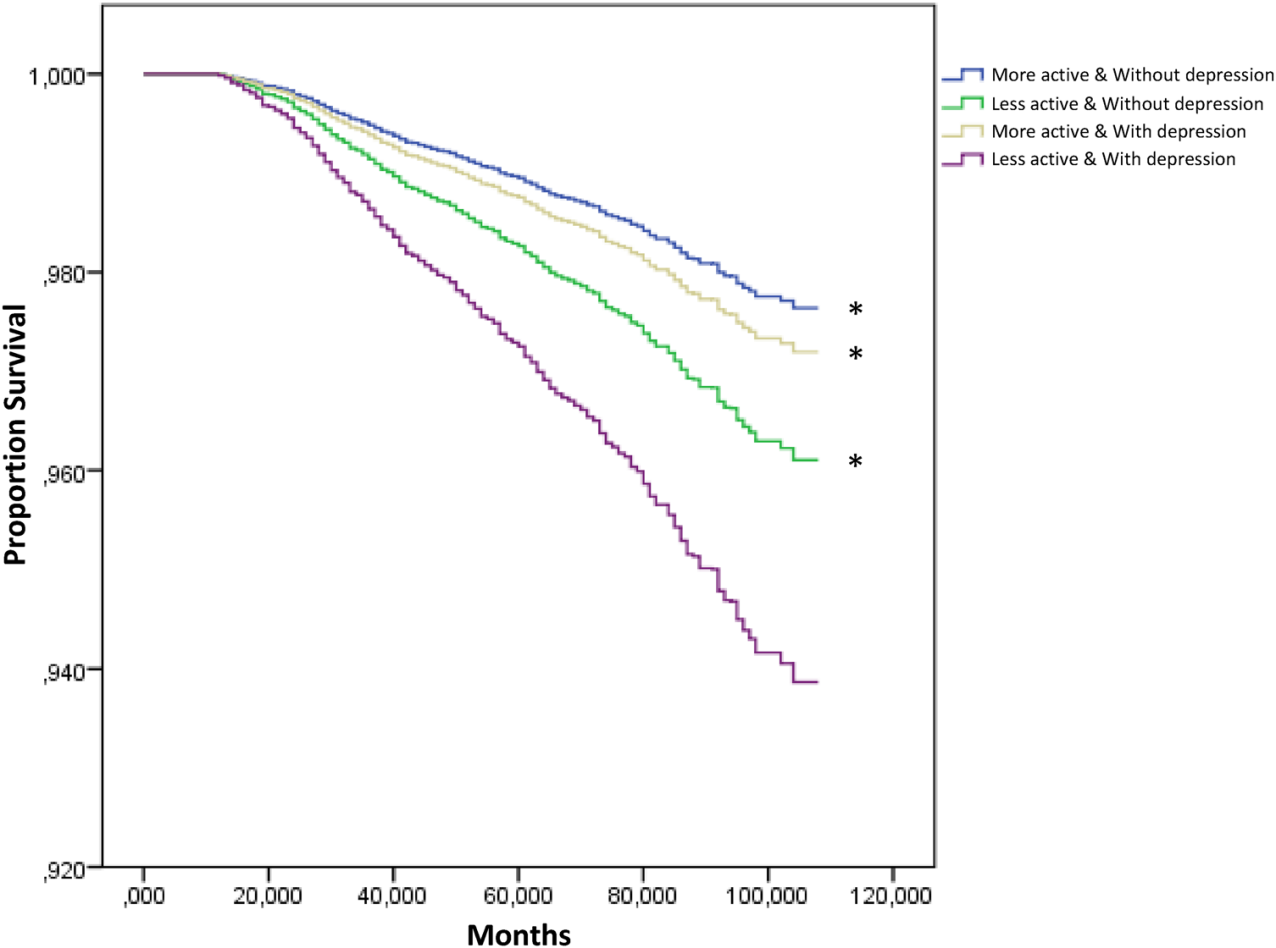
Survival curves for all-cause mortality according to physical activity group and depression status. Data are representative of non-institutionalized American population. Less active: Performed < 600 MET-min/week; More active: Performed ≥ 600 MET-min/week; With depression: Scored ≥ 10 in the PHQ-9; Without depression: Scored < 10 in the PHQ-9. Curves are adjusted by age, sex, race/ethnicity, annual household income, educational level, alcohol consumption, smoking status, BMI, arterial hypertension, dyslipidemia, and diabetes. *Significant difference with Less active & with depression group.

When the joint association of depression and PA was analyzed in relation to the risk of all-cause mortality, those who were more active without depression had the lowest risk of death compared to those who were less active and with depression, HR 0.38 (95% CI 0.28, 0.52, p < 0.001). Those who were less active without depression, and those who were more active with depression, also had a lower risk of death compared to those who were less active and with depression, 0.63 HR (95% CI 0.46, 0.85, p = 0.003), and 0.45 HR (95% CI 0.22, 0.91, p = 0.026), respectively (Fig. 2). These HRs were adjusted by age, sex, race/ethnicity, annual household income, educational level, alcohol consumption, smoking status, BMI, arterial hypertension, dyslipidemia, and diabetes.

## Discussion

This study provided evidence that performing at least 150 min/week of moderate to vigorous PA was associated with reduced odds for depression among an American population aged 50 and older. Furthermore, among those with depression, performing 150 min/week of moderate to vigorous PA was associated with a 55.1% reduced risk of all-cause mortality compared to those who performed less PA.

The beneficial effect of PA in depression prevention has been analyzed in-depth through specific systematic reviews, concluding that PA may prevent depression^10,11,29^. Nevertheless, only a few studies were focused on older people^15,16,30–33^, and in addition, only two of those studies conducted in European and Asian populations analyzed the relationship between depression and PA assessed as a dose (combined amount and intensity)^15,16^. However, controversial findings were reported by these two studies. Mc Dowell et al. conducted a study with more than 7800 participants and supported an association between meeting PA guidelines and lower odds of depression after adjusting the results by age, sex, and BMI (OR 0.56, 95% CI 0.47, 0.66)^16^. On the other hand, Wang et al. did not find an association between meeting PA guidelines (performing 600–2249 MET-min/week of PA) and lower odds of depression (OR 1.02, 95% CI 0.78, 1.33), but found an association between performing more than 2250 MET-min/week of PA and higher odds of depression (OR 1.22, 95% CI 1.01, 1.47)^15^. As Wang et al. discussed, the association of very high levels of PA with a higher risk of depression may be due to the purpose of PA, since higher frequency, longer duration, and larger volume of heavy-labor work may indicate lower household income, which leads to increasing the risk of depression^15^. The findings of our study are in line with those of Mc Dowell, reinforcing the idea that meeting PA guidelines can help prevent depression among persons older than 50 years.

As we mentioned above, increasing evidence shows a beneficial effect of PA in depression prevention^10,11,29^. However, the causality and direction of this association have been discussed in the literature, suggesting that PA may protect against depression, and/or depression may result in decreased PA. This could be a source of concern in ascertaining the role of PA in depression prevention. Nevertheless, a meta-analysis of prospective studies, and other recent study using bidirectional mendelian randomization provide evidence to establish a causal relationship between PA and a reduced risk for depression^11,34^.

Previous studies have analyzed the association between meeting the PA recommendations in adults and all-cause mortality, establishing that those meeting the recommendations have a 40% decreased risk of death^9,17^. Other previous studies also have ascertained a positive association between depression and all-cause mortality^18^. The results of our study are consistent with those previous studies, establishing that 150 min of moderate to vigorous PA can be a protective factor against all-cause mortality and that having depression is associated with a higher all-cause mortality risk. However, the present study also analyzes the combined effects of meeting the PA recommendations and depression status with the risk of all-cause mortality among persons older than 50 years. As could be expected, those without depression in the more-active group had the lowest HR for all-cause mortality compared with those with depression in the less-active group. Interestingly, those with depression in the more-active group had a lesser HR than those without depression in the less-active group, compared with the reference group (with depression and less active). This fact reveals that PA could counteract the higher mortality risk due to depression.

Analyzing the influence of PA as a dose (combined amount and intensity) in relation to depression and all-cause mortality, and not only as frequency, as in other studies^22,31,32^, is essential. However, it could be interesting to analyze whether the type of PA influences the relationship of PA's preventive role against depression and whether the combined effect of PA and depression status on all-cause mortality is PA-type dependent. Other studies have elucidated the relationship between PA and mortality, finding that it is dependent on the type of PA^17^. Therefore, differentiating at least between endurance and resistance activities in PA quantification may determine if any type of PA is more protective than others against depression. One previous study has shown that regular flexibility, and no other type of exercise, such as muscular strength or walking, was independently related to depression prevention^22^. However, as mentioned above, in this study, the assessment of PA only as frequency may not show the real role of each PA type in depression prevention^22^. Maybe future studies could shed some light on this issue.

To the best of our knowledge, this is the first study that analyzed the joint association between PA and depression with all-cause mortality in a representative sample of the American population aged 50 and older. However, several limitations should be acknowledged in our study. First, regarding the association of PA and depression, the cross-sectional analysis does not allow us to establish a causal, temporal link. Second, although data collection about PA has been carried out by trained interviewers, the use of self-reported information could be subject to bias^35^. Third, depression status was only assessed once (at baseline), and it was not possible to consider the course of depression. Fourth, to increase the statistical power, only two subgroups of total PA were used to test in combination with depression status, its joint association with mortality. Fifth, only non-institutionalized adults were included in this analysis, so the results can only be applied to this population.

## Conclusions

In summary, performing 150 min/week of moderate to vigorous PA is associated with reduced odds for depression and seems to be a preventive factor against the increased all-cause mortality risk due to depression. From a population health perspective, promoting moderate to vigorous PA for at least 150 min/week among Americans aged over 50 years with depression may be an important health-promotion strategy that can reduce the increased all-cause mortality risk associated with depression.

## Supplementary Information

Supplementary Tables.
