# Supplementary material for: A cross-sectional analysis of the association between physical activity, depression, and all-cause mortality in Americans over 50 years old
Source: Sci Rep. 2022 Feb 10;12:2264. doi: 10.1038/s41598-022-05563-7 (PMC8846564; doi:10.1038/s41598-022-05563-7)
Supplement: Supplementary file 1 — Supplementary Tables. [file 41598_2022_5563_MOESM1_ESM.docx]

|  | **Physical activity level (MET-min/week)** | | |
| --- | --- | --- | --- |
|  | **Low**  **(<600)** | **Medium**  **(600 - 1200)** | **High**  **(>1200)** |
| N | 43.1 % | 14.5 % | 42.4 % |
| Unadjusted | 1.00 (ref) | 0.54 (0.38, 0.78)* | 0.43 (0.33, 0.55)* |
| Age-adjusted | 1.00 (ref) | 0.52 (0.36, 0.75)* | 0.17 (0.14, 0.21)* |
| Multivariable-adjusted Model A | 1.00 (ref) | 0.59 (0.41, 0.84)* | 0.49 (0.38, 0.63)* |
| Multivariable-adjusted Model B | 1.00 (ref) | 0.66 (0.46, 0.95)* | 0.53 (0.41, 0.70)* |

**Supplementary Table 1:** Odds ratio (95% CI) for depression according to physical activity levels. Data are representative of non-institutionalized American population. Model A is adjusted by age, sex, race/ethnicity, annual household income, and educational level. Model B is additionally adjusted by alcohol consumption, smoking status, BMI, arterial hypertension, dyslipidemia, and diabetes. ***** Significant differences with Low group (p<0.05).

|  | **Physical activity (MET-min/week)** | |  |
| --- | --- | --- | --- |
|  | **Less active (<600)** | **More active (≥600)** | ***p-*Value** |
| **Physical activity at work/domestic** |  |  |  |
| N | 69.5 % | 30.5 % |  |
| Unadjusted | 1.00 (ref) | 0.80 (0.59, 1.07) | 0.125 |
| Age-adjusted | 1.00 (ref) | 0.73 (0.55, 0.99) * | 0.041 |
| Multivariable-adjusted Model A | 1.00 (ref) | 0.78 (0.58, 1.06) | 0.108 |
| Multivariable-adjusted Model B | 1.00 (ref) | 0.82 (0.60, 1.11) | 0.196 |
| **Physical activity in leisure time** |  |  |  |
| N | 67.8 % | 32.2 % |  |
| Unadjusted | 1.00 (ref) | 0.31 (0.22, 0.44) * | <0.001 |
| Age-adjusted | 1.00 (ref) | 0.30 (0.21, 0.42) * | <0.001 |
| Multivariable-adjusted Model A | 1.00 (ref) | 0.42 (0.30, 0.60) * | <0.001 |
| Multivariable-adjusted Model B | 1.00 (ref) | 0.47 (0.32, 0.67) * | <0.001 |
| **Physical activity in transport/travel** |  |  |  |
| N | 88.7 % | 11.3 % |  |
| Unadjusted | 1.00 (ref) | 0.77 (0.52, 1.15) | 0.198 |
| Age-adjusted | 1.00 (ref) | 0.73 (0.49, 1.09) | 0.124 |
| Multivariable-adjusted Model A | 1.00 (ref) | 0.71 (0.48, 1.04) | 0.078 |
| Multivariable-adjusted Model B | 1.00 (ref) | 0.79 (0.53, 1.16) | 0.225 |

**Supplementary Table 2:** Odds ratio (95% CI) for depression according to physical activity performed in different domains (work/domestic, leisure time, transport/travel). Data are representative of non-institutionalized American population. Model A is adjusted by age, sex, race/ethnicity, annual household income, and educational level. Model B is additionally adjusted by alcohol consumption, smoking status, BMI, arterial hypertension, dyslipidemia, and diabetes. ***** Significant differences between Less-active and More-active groups.
